# Erythrocyte-Bound Apolipoprotein B in Relation to Atherosclerosis, Serum Lipids and ABO Blood Group

**DOI:** 10.1371/journal.pone.0075573

**Published:** 2013-09-19

**Authors:** Boudewijn Klop, Gert-Jan M. van de Geijn, Sarah A. Bovenberg, Noëlle van der Meulen, Jan Willem F. Elte, Erwin Birnie, Tjin L. Njo, Hans W. Janssen, Addy van Miltenburg, J. Wouter Jukema, Manuel Castro Cabezas

**Affiliations:** 1 Department of Internal Medicine, Diabetes and Vascular Center, Sint Franciscus Gasthuis, Rotterdam, The Netherlands; 2 Department of Clinical Chemistry, Sint Franciscus Gasthuis, Rotterdam, The Netherlands; 3 Department of Statistics and Education, Sint Franciscus Gasthuis, Rotterdam, The Netherlands; 4 Department of Cardiology, Sint Franciscus Gasthuis, Rotterdam, The Netherlands; 5 Department of Cardiology, Leiden University Medical Center, Leiden, The Netherlands; Medical University of Graz, Austria

## Abstract

**Introduction:**

Erythrocytes carry apolipoprotein B on their membrane, but the determining factors of erythrocyte-bound apolipoprotein B (ery-apoB) are unknown. We aimed to explore the determinants of ery-apoB to gain more insight into potential mechanisms.

**Methods:**

Subjects with and without CVD were included (N = 398). Ery-apoB was measured on fresh whole blood samples using flow cytometry. Subjects with ery-apoB levels ≤0.20 a.u. were considered deficient. Carotid intima media thickness (CIMT) was determined as a measure of (subclinical) atherosclerosis.

**Results:**

Mean ery-apoB value was 23.2% lower in subjects with increased CIMT (0.80±0.09 mm, N = 140) compared to subjects with a normal CIMT (0.57±0.08 mm, N = 258) (P = 0.007, adjusted P<0.001). CIMT and ery-apoB were inversely correlated (Spearman’s r: –0.116, P = 0.021). A total of 55 subjects (13.6%) were considered ery-apoB deficient, which was associated with a medical history of CVD (OR: 1.86, 95% CI 1.04–3.33; adjusted OR: 1.55; 95% CI 0.85–2.82). Discontinuation of statins in 54 subjects did not influence ery-apoB values despite a 58.4% increase in serum apolipoprotein B. Subjects with blood group O had significantly higher ery-apoB values (1.56±0.94 a.u.) when compared to subjects with blood group A (0.89±1.15 a.u), blood group B (0.73±0.1.12 a.u.) or blood group AB (0.69±0.69 a.u.) (P-ANOVA = 0.002).

**Conclusion:**

Absence or very low values of ery-apoB are associated with clinical and subclinical atherosclerosis. While serum apolipoprotein B is not associated with ery-apoB, the ABO blood group seems to be a significant determinant.

## Introduction

The cardiovascular complications of atherosclerosis remain a major health problem in the general population. Atherosclerosis is a slowly progressive disease, induced by numerous risk factors contributing to lipid deposition, inflammation and atherothrombosis [1]. Apolipoprotein (apo) B is the structural protein of the atherogenic lipoproteins including chylomicrons and their remnants, VLDL, IDL and LDL. Large studies have shown that the concentration of serum apo B is a strong predictor of CVD [2]–[4]. Lipoproteins are found in the fluid phase where they are metabolized and transported to specific organs. However, there is also evidence of a marginated pool of apo B containing lipoproteins attached to the endothelium and to circulating blood cells [5], [6]. Close interaction exists between circulating leukocytes and apo B containing lipoproteins as has been demonstrated in human studies [7]–[9].

Erythrocytes represent the largest blood cell population and make up more than 99% of the total cellular space in blood [10]. It has been suggested that the exchange of cholesterolesters between LDL and erythrocyte membranes may be substantial, which can only be explained by binding of LDL to erythrocytes and not by accidental collision [11], [12]. Recently, it was shown that erythrocytes may contribute to reverse cholesterol transport with impairment of reverse cholesterol transport in anemic mice [13]. In a relatively small pilot study, binding of apolipoprotein (apo) B containing lipoproteins to erythrocytes (ery-apoB) was proposed as a protective factor for cardiovascular disease (CVD) [14]. In this study no clear correlation was observed between serum apo B concentrations and ery-apoB and no significant determinants of ery-apoB were detected.

We investigated the association of ery-apoB with carotid intima media thickness (CIMT) and CVD in a larger study population. In addition, we tested whether statins and serum apo B concentrations influence ery-apoB values in a separate group of subjects. Finally, we explored the association between the ABO blood group system and ery-apoB values.

## Materials and Methods

### Subjects

Participants were recruited from the outpatient clinics of the Diabetes Vascular Center and the department of Cardiology, Sint Franciscus Gasthuis in Rotterdam, for the measurement of ery-apoB. The inclusion was carried out between July 2009 and February 2013. Both subjects with and without a history of CVD were included since we expected an atheroprotective effect from ery-apoB. A history of CVD was defined as the presence of at least one of the following conditions before inclusion: a myocardial infarction, angina pectoris based on clinical characteristics, documented coronary artery disease based on a coronary angiogram, a cerebral infarction or the presence of peripheral artery disease. Exclusion criteria were age <18 years or the use of any experimental medication within 6 months before participation. Anthropometric characteristics, e.g. weight, length, BMI, waist circumference and blood pressure measurements were recorded. Carotid ultrasound scans were carried out to measure carotid intima media thickness (CIMT) using the ART-LAB (Esaote, Italy), which has been described in detail previously [14].

ABO blood groups were obtained from the clinical registry system and if not available, participants were asked to have their ABO blood group determined in our hospital on a separate occasion.

A separate group of subjects who used statins for primary cardiovascular prevention discontinued their statin therapy to investigate the effect of statins and subsequent changes in serum apo B concentrations on ery-apoB values. Participants using statins, but not other lipid lowering drugs, visited the outpatient clinic for baseline measurements including ery-apoB and they were asked to discontinue statin therapy for 6 weeks followed by a second visit including a second blood draw with the measurement of ery-apoB. The subjects were fasting during both visits. This substudy was registered at Clinicaltrials.gov (NCT01634906).

All subjects provided written informed consent. The studies were approved by the independent Regional Medical Ethical Committee Rotterdam, Maasstad Hospital, the Netherlands.

### In vivo measurement of ery-apoB

Blood samples for the determination apo B bound to erythrocytes were obtained in tubes containing 5.4 mg K2 EDTA (Becton Dickinson, Plymouth, UK). The staining procedure was started within one hour after venipuncture. The method has been described in detail previously [7], [14]. To avoid interference of serum lipoproteins, the samples were washed three times in PBS supplemented with 0.5% BSA (PBS-BSA). The erythrocytes were incubated with a polyclonal goat antibody directed against human apo B (catalogue no. AB742; Millipore, Billerica, MA, USA) for 30 minutes in the dark on ice. Subsequently, the erythrocytes were washed with PBS-BSA and incubated with a rabbit anti-goat (RAG) antibody conjugated with FITC (Nordic Immunological Laboratories, Tilburg, the Netherlands) for another 30 minutes in the dark on ice. As a control for background staining, each sample was simultaneously stained in parallel without anti-apo B antibodies, but with RAG-FITC. Samples were kept in the dark on ice until measurement. A total of 5000 erythrocytes per sample were analysed by flow cytometry using an Epics XL-flow cytometer (Beckman Coulter, Miami, Florida, USA). An FC500 flow cytometer (Beckman Coulter) was used in case of the statin withdrawal sub-study. Before each use, the optics and settings of the flow cytometer were checked with Flow-Check and Flow-Set beads (Beckman Coulter). Identical flow cytometric settings were used for the complete study.

### Laboratory measurements

All clinical and haematological chemistry measurements were carried out on freshly drawn blood samples and analysed in the Department of Clinical Chemistry, Sint Franciscus Gasthuis. Baseline glucose, plasma cholesterol, HDL-C, triglycerides, C-reactive protein, creatine kinase, ALAT and ASAT were measured using the LX20 and DxC analyzers (Beckman Coulter). LDL-C values were calculated using the Friedewald formula. Apo A-I and apo B were determined by nephelometry using an IMMAGE instrument with commercially available kits (Beckman Coulter). Blood cell counts were determined automatically using LH750 and DxH800 analyzers (Beckman Coulter). Thyroid function (TSH) was determined by TSH measurement (Immulite 2500; Siemens, Healthcare Diagnostics, Deerfield, Illinois, USA). The ABO blood group was determined by standard procedures using agglutination techniques (Galileo Echo; Immucor Gamma, Heppignies, Belgium).

### Statistics

The binding of apo B on erythrocytes was expressed as the mean difference between fluorescence intensity of apo B-staining and control background staining in arbitrary units (a.u.). The mean fluorescence intensity reflects the amount of apo B per erythrocyte. For statistical analysis, CIMT was defined as the mean of the six individual measurements. Subjects were divided into two groups: a normal CIMT (<0.70 mm) or increased CIMT (*≥*0.70 mm). Linear regression analysis (backward stepwise) was used to test the differential impact of ery-apoB on CIMT adjusted for multiple variables. The variables included were age, gender, a medical history of CVD, BMI, HDL-C, T2DM and the following interaction effects: CIMT × gender, CIMT × CVD, CIMT × T2DM, CIMT × age, CIMT × BMI, CIMT × HDL-C, BMI × HDL-C, BMI × T2DM, CVD × age, T2DM × age, T2DM × HDL-C, HDL- × gender, CVD × HDL-C, CVD × BMI. Subjects with ery-apoB levels ranging from undetectable to 0.20 a.u. were considered ery-apoB deficient. Since leukocyte count and HDL-C were significantly different between ery-apoB deficient and sufficient subjects, the impact of ery-apoB deficiency on CVD risk was adjusted for leukocyte count and HDL-C using multiple binary logistic regression analysis.

A power calculation demonstrated that at least 53 subjects were necessary to demonstrate a significant difference of 0.25±0.64 a.u. in ery-apoB after statin withdrawal (power 80%, alpha 0.05 two-sided). The subjects with ABO blood group measurements were divided into tertiles based upon their ery-apoB values to test the distribution of the ABO blood group between the respective tertiles with the Chi-square test. Differences in continuous variables between two groups were tested with the independent Student’s *t*-test. One-way ANOVA with LSD as post-hoc analysis was used for comparing multiple groups. The LSD test was not corrected for multiple comparisons. However, we performed the LSD test only when the overall ANOVA resulted in a P value less than 0.05.

Skewed variables, which included ery-apoB, triglycerides and C-reactive protein, were logarithmically transformed before analysis, but non-transformed data are shown in the text and tables. Correlations were obtained using the bivariate Spearman’s correlation. All statistical analyses were performed using PASW statistics version 18.0 (IBM SPSS Statistics, New York, United States). A P-value of <0.05 (two sided) was regarded as statistical significant.

## Results

### Carotid intima media thickness and ery-apoB

A total of 409 subjects were included in the study. Levels of ery-apoB were missing in 11 subjects due to technical failures and they were left out of the analysis. A total of 258 subjects (64.8%) showed a normal CIMT and 140 (35.2%) had an increased CIMT. Baseline characteristics of these subjects are shown in Table 1. Subjects with an increased CIMT were older and were more frequently male and obese. They had more frequently a medical history of CVD and used more often statins. The serum apo B concentration was similar between the two groups. Ery-apoB was significantly lower in subjects with increased CIMT (0.89±0.83 a.u.) compared to subjects with a normal CIMT (1.16±0.92; P = 0.007). The difference between the two groups remained significant after adjustment for multiple variables (0.71±0.08 a.u. versus 0.60±0.14 a.u.; P<0.001). A reverse correlation existed between CIMT and ery-apoB (Spearman’s r: −0.116, P = 0.021).

**Table 1:** Characteristics of subjects based on carotid intima media thickness and on deficiency of ery-apoB (ery-apoB ≤0.20 a.u.) and ery-apoB sufficiency (ery-apoB >0.20 a.u.).

	CIMT <0.70 (N = 258)	CIMT *≥* 0.70 (N = 140)	P-value	Ery-apoB deficiency(N = 55)	Ery-apoB sufficiency(N = 343)	P-value
Age (years)	54.2±11.7	65.8±8.7	<0.001	60.8±13.3	58.0±11.9	0.11
Male gender (n, %)	125 (48.4)	94 (67.1)	<0.001	31 (56.4)	187 (54.4)	0.78
History of T2DM (n, %)	31 (12.0)	35 (25.0)	0.001	13 (23.6)	58 (16.9)	0.22
History of CVD (n, %)	99 (38.4)	91 (65.0)	<0.001	33 (60.0)	153 (44.6)	0.03
Use of statins (n, %)	100 (43.4)	97 (69.3)	<0.001	35 (63.6)	173 (50.7)	0.08
BMI (kg/m^2^)	26.4±4.5	27.9±4.6	0.001	27.5±4.8	27.0±4.6	0.41
CIMT (mm)	0.57±0.08	0.80±0.09	<0.001	0.673±0.132	0.646±143	0.19
Hemoglobin (mmol/l)	8.8±0.7	8.8±0.9	0.72	8.8±0.86	8.8±0.7	0.89
Erythrocytes (*10^12^/l)	4.6±0.4	4.6±0.4	0.33	4.6±0.4	4.6±0.4	0.80
Leukocytes (*10^9^/l)	6.5±1.9	6.9±1.7	0.04	7.2±1.6	6.5±1.8	0.02
Platelets (*10^9^/l)	232±54	237±59	0.47	244±58	232±56	0.13
C-reactive protein (mg/l)	2.7±3.0	3.3±3.0	0.10	3.6±3.9	2.7±2.8	0.06
Total cholesterol (mmol/l)	4.9±1.2	4.7±1.1	0.12	4.7±1.1	4.9±1.2	0.35
LDL-C (mmol/l)	2.9±1.1	2.7±1.0	0.19	2.7±1.0	2.8±1.1	0.51
HDL-C (mmol/l)	1.44±0.42	1.31±0.38	0.01	1.26±0.36	1.41±0.42	0.01
Triglycerides (mmol/l)	1.44±1.01	1.68±0.93	0.03	1.70±0.97	1.50±0.99	0.16
Apolipoprotein B (g/l)	0.93±0.29	0.93±0.25	0.96	0.92±0.26	0.93±0.27	0.78
Apolipoprotein AI (g/l)	1.56±0.32	1.50±0.24	0.10	1.48±0.25	1.55±0.31	0.14

Abbreviations: BMI  =  body mass index; CIMT  =  carotid intima media thickness.

### Ery-apoB deficiency and CVD risk

A total of 55 subjects (13.8%) were deficient for ery-apoB with undetectable to very low (≤0.20 a.u.) ery-apoB values. The remaining subjects were classified as ery-apoB sufficient (N = 343). Characteristics of the two groups are shown in Table 1. The two groups were comparable, except for a higher leukocyte count and lower HDL-C in the ery-apoB deficient group. The risk of CVD was significantly higher in the ery-apoB deficient group compared to the ery-apoB sufficient group (60.0% vs 44.6%, P = 0.034).

Ery-apoB deficiency was associated with an almost two-fold increased prevalence of a medical history of CVD (OR 1.86; 95% CI 1.04–3.33). However, after adjustment for HDL-C and leukocytes the association did not reach statistical significance (adjusted OR 1.55; 95% CI 0.85–2.82).

### The effect of statin use on ery-apoB

**Figure 1:**
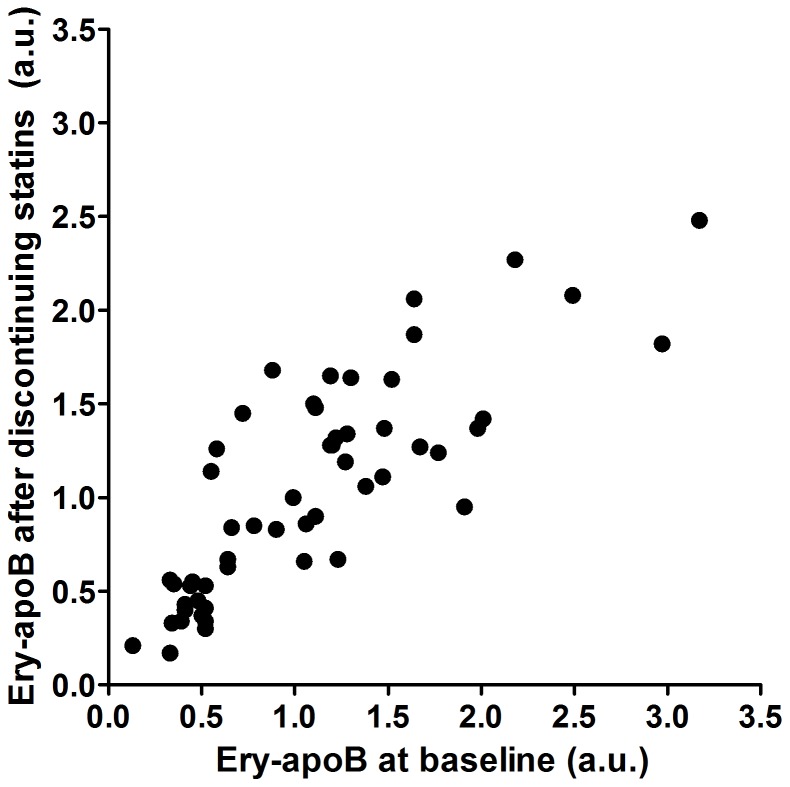
Discontinuation of statin therapy did not affect erythrocyte-bound apolipoprotein B (ery-apoB). Ery-apoB was measured in subjects using statins (N = 54) at baseline and after discontinuing statin therapy for 6 weeks. Individual ery-apoB levels remained fairly stable during the 6 weeks of follow-up since ery-apoB at baseline was strongly correlated to ery-apoB after discontinuing statin therapy for 6 weeks (Spearman r: 0.828; P<0.001).

The impact of statin withdrawal on serum apo B and ery-apoB values were tested in a group of 54 subjects who discontinued their statin therapy for 6 weeks. As expected, total cholesterol, LDL-C, triglycerides and serum apo B increased, whereas creatine kinase and aspartate aminotransferase decreased 6 weeks after statin withdrawal (Table S1). Ery-apoB remained unchanged before and after discontinuation with a mean change in ery-apoB of -0.04±0.39 (P = 0.49) after statin withdrawal. Ery-apoB at baseline was strongly correlated to ery-apoB after discontinuing statin therapy for 6 weeks (Spearman r: 0.828; P<0.001) (Figure 1). Ery-apoB did not correlate with serum apo B before (Spearman r: −0.025; P = 0.857) nor after 6 weeks of statin withdrawal (Spearman r: 0.038; P = 0.786).

### Relationship between ABO blood group and ery-apoB

**Figure 2:**
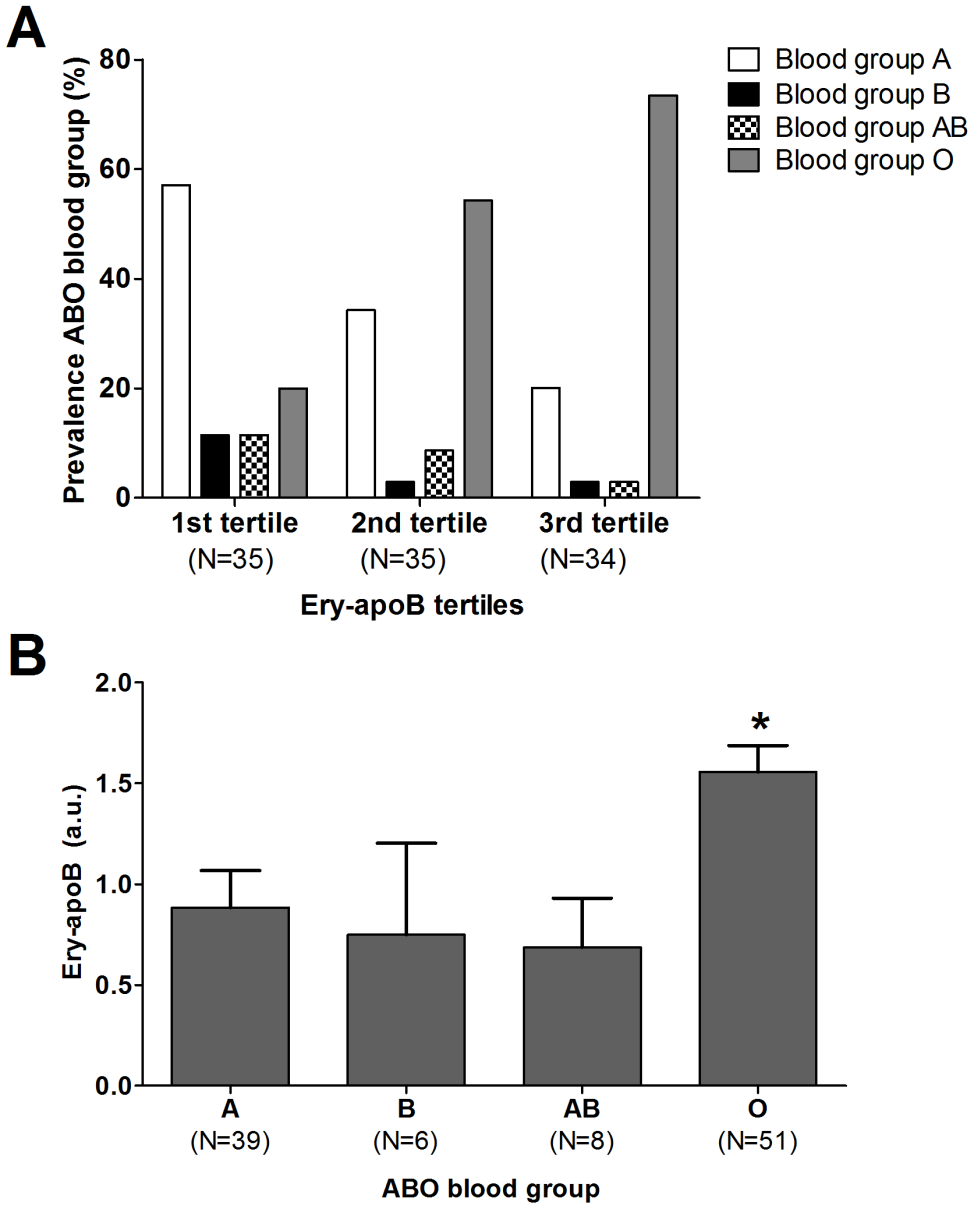
The association between erythrocyte-bound apolipoprotein B (ery-apoB) and ABO blood group phenotypes. The prevalence of the ABO blood group phenotypes per tertile are shown (A). Tertiles are based upon ery-apoB. The first tertile represents the group with the lowest ery-apoB, whereas the third tertile represents the subjects with the highest ery-apoB. The prevalence of ABO blood group phenotypes was significantly different between the three groups (P = 0.002). Ery-apoB levels were almost two-fold increased in subjects with blood group O when compared to subjects with blood group A, B or AB (P-ANOVA <0.001) (B). *P<0.05 when compared to subjects with blood group A, B or AB.

The ABO blood group was included in the analyses to evaluate factors related to ery-apoB. The ABO blood group was available in only 104 subjects of the original cohort. Subjects were divided into tertiles based on their ery-apoB value. The mean ery-apoB value for each of the tertiles were 0.17±0.11 a.u., 0.98±0.36 a.u. and 2.46±0.76 a.u., respectively. The prevalence of CVD was lowest in the third tertile (55.9%) when compared to the first (82.3%) and second tertile (82.3%) with a similar trend for the use of statins (Table S2). The prevalence of blood group A, B and AB were markedly higher in the first tertile when compared to the third tertile, whereas blood group O was much more prevalent in the second and third tertile when compared to the first tertile (P = 0.002) (Figure 2A).

Ery-apoB levels were also analyzed when subjects were divided according to the ABO blood group phenotype. Subjects with blood group A showed higher LDL-C concentrations when compared to subjects with blood group O (Table 2). Mean ery-apoB levels were 1.56±0.94 a.u. in subjects with blood group O (N = 51), which was almost two-fold higher when compared to subjects with blood group A (N = 39) (0.89±1.15 a.u.; P<0.001), blood group B (N = 6) (0.73±1.12 a.u.; P = 0.02) or blood group AB (N = 8) (0.69±0.69 a.u.; P = 0.015) (Figure 2B).

**Table 2:** Characteristics of a select group of subjects with measurements of erythrocyte-bound apolipoprotein B (ery-apoB) and ABO blood group phenotype.

	A (N = 39)	B (N = 6)	AB(N = 8)	O (N = 51)	P-value
Age (years)	61.7±9.5	56.7±16.7	67.6±8.6	61.7±9.3	0.23
Male gender (n, %)	24 (61.5)	3 (50.0)	5 (62.5)	31 (60.8)	0.96
History of T2DM (n, %)	9 (23.1)	0 (0.0)	3 (37.5)	10 (19.6)	0.38
History of CVD (n, %)	27 (69.2)	4 (66.7)	7 (87.5)	39 (76.5)	0.67
Use of statins (n, %)	27 (69.2)	5 (83.3)	8 (100.0)	41 (80.3)	0.30
BMI (kg/m^2^)	27.0±5.2	28.3±4.2	27.9±3.8	27.1±5.0	0.92
Hemoglobin (mmol/l)	8.8±0.8	8.9±0.6	8.5±1.1	8.9±0.8	0.72
Erythrocytes (*10^12^/l)	4.6±0.4	4.7±0.4	4.4±0.6	4.6±0.4	0.53
Leukocytes (*10^9^/l)	7.1±1.6	6.1±1.2	6.8±1.0	6.5±1.6	0.22
Platelets (*10^9^/l)	235±56	237±48	252±63	226±45	0.55
C-reactive protein (mg/l)	3.4±3.9	1.8±1.0	2.7±2.5	2.2±1.8	0.34
Ery-apoB (a.u.)	0.89±1.15*	0.73±1.12*	0.69±0.69*	1.56±0.94***	<0.001
Total cholesterol (mmol/l)	4.7±1.0	4.4±1.3	4.1±0.8	4.4±0.9	0.18
LDL-C (mmol/l)	2.7±0.9*	1.9±1.0	2.3±0.6	2.2±0.8**	0.04
HDL-C (mmol/l)	1.34±0.37	1.63±0.65	1.30±0.41	1.42±0.46	0.41
Triglycerides (mmol/l)	1.65±0.85	1.88±1.83	1.09±0.60	1.76±1.11	0.36
Apolipoprotein B (g/l)	0.91±0.24	0.75±0.24	0.80±0.18	0.82±0.19	0.12
Apolipoprotein AI (g/l)	1.57±0.29	1.57±0.27	1.52±0.30	1.60±0.35	0.90

Subjects were divided according to ABO blood group phenotype.

* Significantly different when compared to blood group O (P<0.05).

** Significantly different when compared to blood group A (P<0.05).

*** Significantly different when compared to blood group A, B or AB (all P<0.05).

## Discussion

Our study confirmed the association of ery-apoB with clinical and subclinical atherosclerosis in an extended study population [14]. Ery-apoB was lower in subjects with increased CIMT and very low to undetectable values of ery-apoB were associated with an increased CVD risk. Recently, it was demonstrated in a mouse model that erythrocytes contribute to reverse cholesterol transport, particularly when the number of HDL particles is low [13]. Erythrocyte mediated reverse cholesterol transport by binding of atherogenic apo B containing lipoproteins could potentially explain the atheroprotective effect of ery-apoB suggested by the present data. In addition, we hypothesize a protective mechanism in which apo B containing lipoproteins bound to erythrocytes are less likely to interact with the endothelium [6], [14]. The observation that anemia is associated with the development of CVD is also in line with our hypothesis [15].

We did not observe any changes in ery-apoB in the group of subjects who discontinued their statin therapy, despite a marked increase in serum apo B. In our comparison between subjects with normal and increased CIMT we did not correct for the use of statins, although subjects with increased CIMT used statins more frequently. In the present study we clearly demonstrated that statins do not influence ery-apoB since there was no change after discontinuation. In addition, there was no association between serum apo B and ery-apoB [14]. We have chosen to study the effects of statin withdrawal for a period of 6 weeks on ery-apoB, because serum concentrations of apo B and LDL-C return to baseline within 4 weeks. In addition, the lifespan of erythrocytes is approximately 12 weeks and half of all erythrocytes would have been renewed within 6 weeks, which potentially may have shown changes in the renewed erythrocyte population. We hypothesize that the adherence of apo B containing lipoproteins to erythrocytes is dynamic with a continuous process of adherence and release of lipoproteins. Therefore, we presume that a period of 6 weeks of statin withdrawal would have been sufficient to study the effects of statins and changes in serum apo B on ery-apoB. Furthermore, we do not think that it would be ethical for patients to refrain from a necessary treatment for a longer period of time.

The exact binding mechanism of apo B containing lipoproteins to erythrocytes has not yet been elucidated. The LDL receptor seems to be a logical candidate to bind apo B containing lipoproteins to erythrocytes but it is not expressed on erythrocytes [11], [12]. Therefore, other binding mechanisms must be operational. The exchange of cholesterolesters between LDL and erythrocyte membranes may be substantial and could not be explained by accidental collision [11], [12]. Here we show for the first time that the ABO blood group system may influence the binding of apo B containing lipoproteins to erythrocytes. Interestingly, we found that ery-apoB levels were almost two-fold increased in subjects with blood group O when compared to subjects with blood group A, B or AB. We have to admit that the ABO blood group was only available in 104 of the total 409 subjects and that the number of subjects with blood group B and AB was low. This could have resulted in a selection bias. However, the results were so striking, that we decided to include them in the current paper. Studies are underway in our laboratory to gain more insight in the relationship between blood groups and ery-apoB. It is tempting to speculate that different carbohydrate groups on the apo B molecule interact with fucose, galactose, N acetyl-galactosamine or N acetyle-glucosamine, which make up the A, B and H antigens [16].

The ABO blood group system has already been associated with plasma cholesterol concentrations more than 40 years ago [17], [18]. Blood group O has been associated with slightly lower cholesterol levels [19], whereas others have found associations between blood group A and elevated plasma concentrations for total cholesterol [20], [21], and LDL-C [21]. We found similar results in our study with elevated plasma LDL-C levels in subjects with blood group A.

Besides with lipids, the ABO blood group system has been associated with CVD. Factors involved in this association are potentially Factor VIII, Von Willebrand Factor, endothelial molecules and platelet proteins [22], [23]. Recently, two large prospective cohort studies plus a meta-analysis demonstrated that the non-O blood group has a higher risk of CVD (RR 1.11; 95%CI 1.05 – 1.18) and that 6.27% of the CVD cases were attributable to inheriting the non-O blood group [24]. Our finding that ery-apoB is associated with both an atheroprotective effect similar to blood group O is in line with these observations. Prospective data regarding ery-apoB and incident CVD are still lacking and the clinical value of ery-apoB as a biomarker in cardiovascular risk prediction needs to be determined.

In conclusion, absence or very low ery-apoB is associated with the presence of clinical and subclinical atherosclerosis. The evaluation of ery-apoB as a clinical useful risk factor for CVD needs to be determined in a large prospective study. Blood group O was strongly associated with high ery-apoB and may provide a new explanation for the association between the ABO blood group system and CVD and plasma lipids. Statins and serum apo B do not affect ery-apoB.

## Supporting Information

Table S1 Changes in ery-apoB and other parameters of subjects who discontinued statin therapy for 6 weeks (N = 54).
(DOC)

Table S2 Characteristics of a select group of subjects with measurements of erythrocyte-bound apolipoprotein B (ery-apoB) and ABO blood group phenotype.
(DOCX)
