# Supplementary material for: Erythrocyte-Bound Apolipoprotein B in Relation to Atherosclerosis, Serum Lipids and ABO Blood Group
Source: PLoS One. 2013 Sep 19;8(9):e75573. doi: 10.1371/journal.pone.0075573 (PMC3777967; doi:10.1371/journal.pone.0075573)
Supplement: Table S1 — Changes in ery-apoB and other parameters of subjects who discontinued statin therapy for 6 weeks (N = 54). (DOC) [file pone.0075573.s001.doc]

**TABLE S1**

Changes in ery-apoB and other parameters of subjects who discontinued statin therapy for 6 weeks (N = 54).

|  | Baseline | After 6 weeks of statin withdrawal | P-value |
| --- | --- | --- | --- |
| Age (years) | 58.8 ± 8.7 | - | - |
| Male gender (N, %) | 29 (53.7%) | - | - |
| BMI (kg/m^2^) | 28.5 ± 5.1 | 28.4 ± 5.1 | 0.45 |
| Waist (cm) | 103.1 ± 13.4 | 104.3 ± 13.9 | 0.09 |
| Total cholesterol (mmol/l) | 4.6 ± 1.0 | 6.9 ± 1.4 | <0.001 |
| LDL-C (mmol/l) | 2.5 ± 0.8 | 4.6 ± 1.1 | <0.001 |
| HDL-C (mmol/l) | 1.40 ± 0.41 | 1.35 ± 0.39 | 0.08 |
| Triglycerides (mmol/l) | 1.72 ± 1.12 | 2.46 ± 2.49 | 0.007 |
| Apolipoprotein B (g/l) | 0.89 ± 0.26 | 1.41 ± 0.32 | <0.001 |
| Apolipoprotein AI (g/l) | 1.41 ± 0.22 | 1.41 ± 0.23 | 0.84 |
| Erythrocytes (*10^12^/l) | 4.85 ± 0.46 | 4.86 ± 0.44 | 0.83 |
| Leukocytes (*10^9^/l) | 6.5 ± 1.7 | 6.3 ± 1.5 | 0.27 |
| C-reactive protein (mg/l) | 2.60 ± 2.74 | 3.26 ± 5.18 | 0.32 |
| ALAT (U/l) | 30.6 ± 11.4 | 28.6 ± 12.6 | 0.10 |
| ASAT (U/l) | 21.8 ± 14.8 | 19.1 ± 12.3 | 0.01 |
| Creatine kinase (U/l) | 122 ± 76 | 106 ± 63 | 0.02 |
| Ery-apoB (a.u.) | 1.08 ± 0.68 | 1.05 ± 0.57 | 0.49 |

Abbreviations: ALAT = alanine aminotransferase; ASAT = aspartate aminotransferase; ery-apoB = erythrocyte bound apolipoprotein B.
