# Supplementary material for: Erythrocyte-Bound Apolipoprotein B in Relation to Atherosclerosis, Serum Lipids and ABO Blood Group
Source: PLoS One. 2013 Sep 19;8(9):e75573. doi: 10.1371/journal.pone.0075573 (PMC3777967; doi:10.1371/journal.pone.0075573)
Supplement: Table S2 — Characteristics of a select group of subjects with measurements of erythrocyte-bound apolipoprotein B (ery-apoB) and ABO blood group phenotype. (DOCX) [file pone.0075573.s002.docx]

**TABLE S2**

Characteristics of a select group of subjects with measurements of erythrocyte-bound apolipoprotein B (ery-apoB) and ABO blood group phenotype. Subjects were divided into tertiles based on their ery-apoB levels.

|  | 1st tertile  (N = 35) | 2nd tertile  (N = 35) | 3rd tertile  (N = 34) | P-value |
| --- | --- | --- | --- | --- |
| Age (years) | 62.8 ± 12.2 | 61.8 ± 8.9 | 60.9 ± 8.3 | 0.75 |
| Ery-apoB (a.u.) | 0.17 ± 0.11^2,3^ | 0.98 ± 0.36^1,3^ | 2.46 ± 0.76^1,2^ | <0.001 |
| Blood group A (n, %) | 20 (57.1) | 12 (34.3) | 7 (20.1) | 0.002 |
| Blood group B (n, %) | 4 (11.4) | 1 (2.9) | 1 (2.9) |  |
| Blood group AB (n, %) | 4 (11.4) | 3 (8.6) | 1 (2.9) |  |
| Blood group O (n, %) | 7 (20.0) | 19 (54.3) | 25 (73.5) |  |
| Male gender (n, %) | 21 (60.0) | 26 (74.3) | 16 (47.1) | 0.07 |
| History of T2DM (n, %) | 9 (25.7) | 6 (17.1) | 7 (20.1) | 0.68 |
| History of CVD (n, %) | 29 (82.3) | 29 (82.3) | 19 (55.9) | 0.01 |
| Use of statins (n, %) | 29 (82.3) | 31 (88.6) | 21 (61.8) | 0.03 |
| BMI (kg/m^2^) | 27.9 ± 4.4 | 27.7 ± 4.9 | 26.0 ± 5.2 | 0.22 |
| Hemoglobin (mmol/l) | 8.9 ± 1.0 | 8.8 ± 0.7 | 8.8 ± 0.7 | 0.98 |
| Erythrocytes (*10^12^/l) | 4.7 ± 0.5 | 4.6 ± 0.3 | 4.5 ± 0.4 | 0.19 |
| Leukocytes (*10^9^/l) | 7.3 ± 1.6^2,3^ | 6.6 ± 1.5^1^ | 6.2 ± 1.2^1^ | 0.01 |
| Platelets (*10^9^/l) | 236 ± 56 | 224 ± 55 | 237 ± 40 | 0.50 |
| C-reactive protein (mg/l) | 3.3 ± 3.3^3^ | 3.0 ± 3.4 | 1.7 ± 1.2^1^ | 0.02 |
| Total cholesterol (mmol/l) | 4.5 ± 0.8 | 4.4 ± 0.9 | 4.6 ± 1.1 | 0.63 |
| LDL-C (mmol/l) | 2.4 ± 0.7 | 2.4 ± 0.9 | 2.5 ± 0.9 | 0.90 |
| HDL-C (mmol/l) | 1.31 ± 0.35 | 1.37 ± 0.42 | 1.52 ± 0.52 | 0.12 |
| Triglycerides (mmol/l) | 1.85 ± 1.18 | 1.69 ± 1.11 | 1.47 ± 0.77 | 0.40 |
| Apolipoprotein B (g/l) | 0.87 ± 0.20 | 0.83 ± 0.21 | 0.84 ± 0.24 | 0.75 |
| Apolipoprotein AI (g/l) | 1.52 ± 0.28 | 1.59 ± 0.31 | 1.64 ± 0.35 | 0.26 |

^1^ Significantly different when compared to the corresponding 1^st^ tertile (P < 0.05)

^2^ Significantly different when compared to the corresponding 2^nd^ tertile (P < 0.05)

^3^ Significantly different when compared to the corresponding 3^rd^ tertile (P < 0.05)
